# From early communication to bimodal vocabulary acquisition: A longitudinal study of hearing children with deaf mothers from infancy to school-age years

**DOI:** 10.1017/S1366728925100308

**Published:** 2025-07-28

**Authors:** Evelyne Mercure, Victoria St. Clair, Laura Goldberg, Kimberley Coulson-Thaker, Mairéad MacSweeney

**Affiliations:** 1 Department of Psychology, Goldsmiths, University of London, London, UK; 2 UCL Institute of Cognitive Neuroscience, University College London, London, UK; 3 Centre for Brain and Cognitive Development, Birkbeck, University of London, London, UK; 4 Hertfordshire Partnership NHS Foundation Trust, Hertfordshire, UK; 5 Essex Partnership University Trust, Essex, UK

**Keywords:** deafness, sign language, vocabulary, bilingualism, sign language, bimodal bilingualism, deaf parenting, infant communication, babbling, gesture

## Abstract

Early language development has rarely been studied in hearing children with deaf parents who are exposed to both a spoken and a signed language (bimodal bilinguals). This study presents longitudinal data of early communication and vocabulary development in a group of 31 hearing infants exposed to British Sign Language (BSL) and spoken English, at 6 months, 15 months, 24 months and 7 years, in comparison with monolinguals (exposed to English) and unimodal bilinguals (exposed to two spoken languages). No differences were observed in early communication or vocabulary development between bimodal bilinguals and monolinguals, but greater early communicative skills in infancy were found in bimodal bilinguals compared to unimodal bilinguals. Within the bimodal bilingual group, BSL and English vocabulary sizes were positively related. These data provide a healthy picture of early language acquisition in those learning a spoken and signed language simultaneously from birth.

## 1 Introduction

Hearing children of deaf adults (also known as CODAs) are likely to be exposed to two languages in different sensory modalities early in life. Their experience includes a signed language (e.g., British Sign Language – BSL), which can be used by the deaf parents themselves and other deaf and hearing people in the child’s environment. A spoken language (e.g., English) can also be used with and around the child by some deaf parents, as well as by hearing relatives and the wider hearing community, including peers and educators. Since signed languages are expressed in the visual modality and spoken languages in the auditory modality, these children can be referred to as ‘bimodal bilinguals’, as opposed to ‘unimodal bilinguals’ who are exposed to two spoken languages. The language experience of hearing bimodal bilinguals varies enormously between children depending on the family composition, the preferred mode of communication of the deaf parents and the family’s integration into deaf and hearing communities.

Whilst research into bilingualism has increased greatly in recent years, the specific case of bimodal bilingualism in hearing children of deaf parents remains scarcely discussed. For *deaf* children, a growing body of evidence suggests that bimodal bilingualism represents a positive approach to education and language acquisition, leading to optimal developmental and educational outcomes (Clark et al., 2020; Davidson et al., 2014; Delcenserie et al., 2024; Hall et al., 2018, 2019; Hoffmeister et al., 2022; Hyde & Punch, 2011; Mitchiner, 2015; Pontecorvo et al., 2023; Rowley et al., 2022). However, studies with *hearing* bimodal bilinguals are scarcer, often based on small samples, present contradictory results and rarely address the earliest phases of language development (Brackenbury et al., 2006; Daniels, 1993; Hofmann & Chilla, 2015; Meadow-Orlans et al., 2004; Schiff & Ventry, 1976; Zaborniak-Sobczak, 2021). This lack of data leaves space for assumptions to be made by researchers and practitioners about simultaneous sign and spoken language acquisition in hearing children. Some fear that learning sign language is likely to result in difficulty learning spoken language (for a discussion, see Chen Pichler et al. (2014)). In a study on lived experiences of deaf parents, many reported facing prejudice and misunderstanding from hearing communities about the language development of their child (St. Clair et al., 2025). Deaf parents reported that many educators and health professionals focussed only on spoken language development in their child, ignoring their sign language development and failing to understand that they were developing bilingually (St. Clair et al., 2025). Other researchers have observed that, in some areas, all children of deaf parents are referred for speech and language pathology services, suggesting that parental deafness can be considered a sufficient reason for referral regardless of the child’s language development (Chen Pichler et al., 2014). In contrast, universal referral is not usually observed for unimodal bilingual children, and this difference suggests that speech and language pathologists and school officials may believe that learning a spoken and a signed language from birth poses risks to language learning, above and beyond other cases of bilingualism. This is an interesting observation given that no scientific evidence has been produced to suggest a greater incidence of language disorders amongst children of deaf than hearing parents (Chen Pichler et al., 2014).

One aspect of language development that has revealed mixed findings in hearing bimodal bilinguals is vocabulary acquisition (see summary in Table 1). Some studies have found that bimodal bilinguals acquire vocabulary similarly to monolinguals. For example, Brackenbury et al. (2006) longitudinally studied one hearing child with deaf parents and found that their English vocabulary was comparable to monolingual norms at 16 and 20 months. The child’s vocabulary in American Sign Language (ASL) was also comparable to norms of deaf children acquiring ASL from their deaf parents. In another study, 21 hearing children with deaf parents between 17 and 30 months were compared to hearing children of hearing parents (Bornstein et al., 1999). Vocabulary size was measured via parental report, and language skills were directly assessed with the Reynell Expressive and Receptive Scales. Responses were accepted in both spoken English and signed ASL. Hearing children of deaf adults did not differ from hearing children of hearing adults on any of these measures. Moreover, Meadow-Orlans et al. (2004) found that 10 out of 15 hearing children with deaf mothers had at least one recognisable sign or word during free play interaction with their mother at 12 months, a proportion similar to that of hearing children with hearing mothers.

Larger vocabulary sizes were also reported in one study with bimodal bilinguals compared to monolingual norms (Daniels, 1993). English vocabulary was measured with the Peabody Picture Vocabulary Test, in 14 hearing bimodal bilingual children between two and 13 years acquiring ASL and English. As a group, the children scored significantly higher than monolingual norms. However, the study did not compare bimodal bilinguals with a control group, which leaves open the possibility that the testing conditions were conducive to better-than-average scores or that the population’s norms had changed over time.

Other studies have reported that hearing bimodal bilinguals may have smaller vocabulary in spoken language compared to monolinguals. For example, Schiff and Ventry (1976) studied a large group of 52 children of deaf adults between 6 months and 12 years. A detailed assessment of each child’s speech and language skills in English revealed that 10 children presented with language acquisition difficulties in the absence of co-occurring factors that could contribute to language learning difficulties, such as child deafness, psychomotor, emotional or neurological factors. Language difficulties included comprehension and vocabulary deficits, indicated by word finding difficulties in spontaneous speech and/or low performance on standardised assessment of vocabulary. It is important to note that this study did not consider that many (if not all) of these children may have been assessed in their second language if they had ASL as their dominant language. In another study, Zaborniak-Sobczak (2021) assessed six hearing Polish bimodal bilinguals between four and 8 years using a standardised assessment. In spoken Polish receptive vocabulary, three of the children scored higher than monolingual norms, while one scored within the average range and two scored lower than average. For spoken Polish expressive vocabulary, one child scored in the average range, while three scored lower than monolingual norms. Similar variability was observed in comparison to monolinguals in six hearing German bimodal bilingual children between 3 and 6 years (Hofmann & Chilla, 2015). While all bimodal bilingual children scored below monolingual average on expressive vocabulary for verbs in spoken German, five out of the six children reached average values on a non-standardised tool designed to assess unimodal bilingual children.

Inconsistent results in studies of vocabulary development in hearing bimodal bilinguals can be explained in part by individual variability, since they are often based on small samples. It is also likely that differences in design, particularly in comparison groups and in construct measurements, can explain part of the inconsistencies. As the Hofmann and Chilla (2015) study demonstrates, it is important to consider whether bimodal bilinguals’ lexicon is compared to monolinguals’ or unimodal bilinguals’ lexicon. When children are acquiring two languages, they experience a reduced amount of exposure to each of their languages compared to monolinguals, and their total lexicon is shared between their two languages. When vocabulary is measured in one language, unimodal bilinguals usually have a smaller vocabulary compared to a monolingual control group or monolingual norms (Byers-Heinlein et al., 2024; Poulin-Dubois et al., 2013; Siow et al., 2023; Stolarova et al., 2016). Because of this general bilingualism effect on vocabulary acquisition, the specific impact of bimodal bilingualism on language development can only be assessed by including a control group of children learning two spoken languages or by comparison with unimodal bilingual norms.

The fact that vocabulary can be measured in different ways in bilinguals adds an extra level of complexity to the study of bilingual vocabulary acquisition. Indeed, while there is vast evidence that unimodal bilinguals have smaller vocabularies than monolinguals when measured in only one of their languages, it is less clear whether unimodal bilinguals’ total or conceptual vocabulary, across their known languages, is similar to that of monolinguals. When a measure of total vocabulary is created by adding the words known in one language to the words known in another language, larger expressive and receptive total vocabularies compared to monolinguals were found in French–English bilinguals between eight and 33 months (Byers-Heinlein et al., 2024), and in 12- to 36-month-old bilinguals in English and various other spoken languages (Siow et al., 2023). In another study, no differences were found at 22-to-30 months of age between English expressive vocabulary in monolinguals and total vocabulary in Spanish and English in bilinguals (Core et al., 2013). However, when measuring conceptual vocabulary as the number of concepts for which the child knows at least one label in either language, bilinguals had similar receptive (Byers-Heinlein et al., 2024; Siow et al., 2023) but smaller expressive vocabularies compared to monolinguals (Byers-Heinlein et al., 2024; Core et al., 2013; Siow et al., 2023). Ideally, vocabulary should be studied in both languages in bilingual children, to assess the size of each lexicon, as well as allowing a measure of conceptual vocabulary.

There are three other methodological matters that must be addressed in order to improve our understanding of bimodal bilinguals’ language development. First, many studies of early vocabulary development rely on parental report tools such as the Communicative Development Inventory (CDI). This tool has been found to have excellent reliability and predictive validity (Reese & Read, 2000), including in a bilingual population (Mancilla-Martinez et al., 2016). However, it may be that some bilingual parents infrequently use one of their languages with their child and are less likely to have an accurate awareness of the child’s vocabulary knowledge in a language that is mainly used outside of the family home, for example, in childcare setting. This caveat also applies to deaf parents who may or may not use spoken language with their child or may not have full access to this language modality. For this reason, it is important to use direct vocabulary assessments in combination with parental reports for a more complete picture of both unimodal and bimodal bilingual children’s vocabulary acquisition trajectory.

Second, many researchers focus only on spoken language when studying bimodal bilinguals, and/or may not make data collection deaf- and sign-language-friendly, both for deaf parents and their signing children. In the case of bimodal bilinguals, it is also unclear how spoken and signed language lexicons relate to each other. Those who fear an interference of signed language with spoken language acquisition (for discussion, see (Chen Pichler et al., 2014; St. Clair et al., 2025) would predict a negative relationship between lexicons in different language modalities. That is, the greater the child’s sign language vocabulary, the smaller the child’s spoken language vocabulary would be. However, the opposite relationship has been observed in deaf children. Indeed, English vocabulary has been found to positively correlate with ASL vocabulary in a group of 56 deaf or hard-of-hearing children between eight and 60 months (Pontecorvo et al., 2023). This positive correlation cannot necessarily be interpreted as suggesting that learning ASL vocabulary is beneficial to learning English vocabulary. Better general language or cognitive skills may be advantageous to vocabulary acquisition in both modalities. Nevertheless, these data clearly suggest that the acquisition of sign language does not harm spoken language vocabulary acquisition in deaf children. To our knowledge, this relationship has not been assessed in hearing bimodal bilinguals. This study aims to fill this gap in the literature.

Third, longitudinal measurements are rarely available in the literature on vocabulary development in bimodal bilinguals, meaning that only a snapshot of children’s language skills, rather than a full picture of their emergence, can be obtained. While it is clear that early communicative skills, babbling and gesturing have strong associations with the transition to formal language (Donnellan et al., 2020; Goldstein et al., 2010; Matthews et al., 2018; McGillion et al., 2017; Morgan et al., 2020; Rowe & Goldin-Meadow, 2009), few studies have focussed on these early building blocks of language in bimodal bilinguals.

The present study aimed to fill gaps in the literature by examining early communication and vocabulary acquisition in children exposed to BSL and spoken English from their deaf mother. Bimodal bilinguals were compared to two control groups: monolingual children exposed exclusively to English and unimodal bilinguals exposed to English and an additional spoken language. These groups were compared across four different time points, covering vocabulary acquisition from its building blocks in infancy all the way to school-age years. Children were initially recruited at 6 months, and a subset was studied longitudinally at 15 months, 24 months and 7 years, using a mixture of direct assessments and parental reports.

At 6 months, the Mullen Scales of Early Learning (MSEL) was administered to assess early communication skills. Questionnaires regarding language exposure were also given to parents. At 15 and 24 months, parents completed the CDI in English for all infants and in BSL for bimodal bilinguals. To obtain a direct measure of vocabulary skills, children were assessed at 7 years with an online battery via the Pearson Clinical Q-Global platform. This battery included the Expressive Vocabulary Test Third Edition (EVT-3), the Vineland Adaptive Behaviour Scales and Raven’s Progressive Matrices. A fluent signer was present at each testing session with a deaf parent to ensure maximal accessibility.

The present study addressed four main aims:

(1) To describe early communicative skills in bimodal bilinguals. Since there is very little literature on this topic, this aim is highly exploratory. Receptive communication skills in infancy are not linked to a specific language and were not expected to differ between groups. However, since they are likely to experience a reduced amount of auditory spoken language, bimodal bilinguals could be less mature than babies of hearing parents in their vocal babbling at 6 months. Finally, given the previously demonstrated impact of sign language experience on communicative hand movements in infancy (Cormier et al., 1998; Petitto et al., 2004; Petitto & Marentette, 1991), bimodal bilinguals were predicted to be more mature in gesture and imitation than monolingual and bilingual infants with hearing parents.
(2) To assess the trajectory of vocabulary acquisition in bimodal bilinguals compared to monolingual and unimodal bilingual children. Consistent with unimodal bilingual literature, it was predicted that bimodal bilinguals may show smaller receptive and expressive English vocabulary sizes than monolinguals. Bimodal bilinguals were expected to perform similarly in receptive and expressive English vocabulary to unimodal bilinguals. Conceptual vocabulary was predicted to be smaller in bilinguals (both unimodal and bimodal) than in monolinguals, in expressive, but not receptive vocabulary.
(3) To test the relationship between spoken and signed language vocabulary within bimodal bilinguals. Based on data of deaf bimodal bilingual children, a positive relationship was predicted between English and BSL vocabulary.
(4) To investigate whether early communicative skills in infancy predict English vocabulary development in toddlerhood and childhood in all infants. This study tests the prediction that early communicative skills, vocal babbling, gesture and imitation skills in infancy predict later development of English vocabulary in a group of children from diverse backgrounds, including unimodal and bimodal bilingual children.

## 2 Methods

### 2.1 Participants

Ninety-one hearing infants between 4 and 8 months contributed data to this study at the first time point. Of these infants, *n* = 31 were bimodal bilinguals with a deaf mother who were exposed to BSL and English, *n* = 27 were unimodal bilinguals exposed to English and another spoken language, and *n* = 33 were monolinguals exposed to English only. Three additional infants were tested but are not included in analyses because the family withdrew from the study (*n* = 1), or the infants were born before 37 weeks of gestation (*n* = 2).

Infants were considered bilingual if their parents reported that they were frequently and regularly exposed to more than one language. A parent interview was conducted with each family using an early version of the Language Exposure Assessment Tool (LEAT) (DeAnda et al., 2016) to gather more information about the language experience of each child. At 6 months, bimodal bilinguals were exposed to BSL on average 56% of the time (standard deviation = 18.9; [17–98]) and to English on average 40% of the time (standard deviation = 21.1 [2–83]). Four bimodal bilingual infants had exposure to an additional spoken language (*n* = 2), an additional signed language (*n* = 1) or both (*n = 1*). Unimodal bilinguals were exposed to English on average 45% of the time (standard deviation = 21.4, [8–87]). Five unimodal bilinguals had exposure to three spoken languages, while one child had exposure to four spoken languages. Additional spoken languages of exposure were Spanish (*n* = 3), Italian (*n* = 3), Greek (*n* = 3), Polish (*n* = 3), German (*n* = 3), Russian (*n* = 3), Bengali (*n* = 3), Cantonese (*n* = 2), Portuguese (*n* = 2), Jamaican Patois (*n* = 1), Norwegian (*n* = 1), Punjabi (*n* = 1), Farsi (*n* = 1), Ukrainian (*n* = 1), Hebrew (*n* = 1), Gujarati (*n* = 1), Swahili (*n* = 1), Catalan (*n* = 1) and Romanian (*n* = 1). There was no significant difference in English exposure between the two groups of bilinguals [*F*(1,58) < 0.01; *p* = 0.957; *η*^2^ = .96]. Infants were born at term (37–42 weeks of gestation), were hearing and sighted, with no history of seizure or serious mental or physical health diagnoses according to their parents.

All families were re-contacted for participation at three further time points, and longitudinal data are presented for *n* = 54 infants around 15 months, *n* = 54 infants around 24 months and *n* = 34 children around 7 years. Groups did not differ in age at any time point (see Table 2). The unimodal bilingual group included more boys than other groups, which resulted in a significant effect of gender at 15 months, but not at other time points (see Table 2). Maternal education did not differ between groups [*x*^2^(6) = 7.51; *p* = .276], but significant differences in household income were observed [*x*^2^(14) = 26.56; *p* = .022] with families with a deaf mother being underrepresented in higher income categories.

At longitudinal time points, parents were presented with the percentage of exposure to each of their child’s languages calculated from LEAT in infancy and asked if this was still representative of their child’s language experience. At 15 and 24 months, most parents of bimodal and unimodal bilinguals reported an increase in English exposure due to changes in childcare settings and increased interaction with siblings or grandparents. At 7 years, all bimodal bilingual children were attending schools that used spoken English, while all unimodal bilinguals were attending schools using English, except for two children who were receiving formal education in their other spoken language.

Bimodal bilinguals were recruited through social media and websites aimed at Deaf communities. Infants with hearing parents were contacted from the Birkbeck Babylab database of volunteers recruited from advertisements at parent-and-baby groups, parenting websites and publications. Deaf families were geographically spread across the United Kingdom, while infants with hearing parents came mostly from the Greater London area. Travel expenses were reimbursed, and a baby t-shirt and certificate of participation were offered to families with infants. Children who took part in the school-age phase of the study received a bookshop voucher. The project details were explained to the parents in English or BSL, depending on the parents’ preferred language. All parents then gave informed written consent prior to participation. The protocol was approved by the Birkbeck, UCL and Goldsmiths Research Ethics Committees and conforms to the Declaration of Helsinki. The authors assert that all procedures contributing to this work comply with the ethical standards of the relevant national and institutional committees on human experimentation and with the *Helsinki Declaration* (https://www.wma.net/what-we-do/medical-ethics/declaration-of-helsinki/doh-oct2008/) of 1975, as revised in 2008.

### 2.2 Procedure

Infants and their caregivers were invited to the Birkbeck Babylab to take part in the study when they were around 6 months. The entire protocol usually required between 1.5 and 3 hours per infant, including resting, napping and feeding time. Infants also took part in a functional near-infrared spectroscopy study (Mercure et al., 2020) and an eye-tracking protocol (Mercure et al., 2018, 2019). Parents were asked to complete questionnaires about language exposure, the child’s developmental history and family demographics. Finally, infants were evaluated with the MSEL by a trained experimenter. According to MSEL procedures, minimal verbal prompts and instructions are given to the child in this age range. In the present study, prompts were given in BSL and English for bimodal bilinguals, and all parents were encouraged to repeat these prompts in the language they use at home. The expressive language scale focusses on oromotor functions (smiling, chewing, swallowing), vocalisations (laughs, coos) and babbling in this age range. In concordance with MSEL procedures, gesture or sign language babbling was not included in this scale. For this reason, in the context of bimodal bilingualism, this scale was renamed ‘vocal expressive language’. Under standard MSEL procedures, some items can be scored by parental report. This means that if a certain behaviour is not observed during the assessment session, the item can still be scored if parents report that their child performs the target behaviour at home. This was likely to cause a problem for the vocal expressive scale because many deaf parents said they were not aware of the details of their child’s babbling, which could have caused an underscoring of this group compared to the infants with hearing parents. For this reason, parental reports were excluded for all infants in the vocal expressive language scale. Instead, all items were coded offline, and the score was based only on observation of vocal babbling and language expression from videos of the MSEL assessment session. Since the vocal expressive items focus on phonological content and syllabic structure of babbling and not yet on word production, video coding did not require the researcher to be fluent in each spoken language children were learning.

Communication Development Inventories were sent by post to the participants when children were around 15 months (Birkbeck Words and Gesture version) and around 24 months (Birkbeck Words and Sentences version). The CDI was sent in English for all infants, as well as in BSL for bimodal bilingual infants. Parents were instructed to fill each questionnaire for each language separately. For example, they were instructed not to include BSL signs that are known when filling the English questionnaire and vice versa. Parents who did not use English with their child were instructed to fill the questionnaire with someone who frequently used the language with their child (i.e., relatives or nursery staff). Parents of unimodal bilingual infants were also sent the CDI in the other languages their child was exposed to (where available). Data from other spoken languages are not presented here, given major differences in the vocabulary lists of the different CDIs. The Words and Gesture CDI sent at 15 months included questions about early communicative skills in addition to the vocabulary checklist. The first section asked parents about early signs of understanding language, such as responding to their own name, expressions that the child seems to understand such as ‘give it to mummy’ and signs of word production such as imitating what adults say. These items were added to an ‘Early language’ composite variable. Moreover, the CDI questionnaire asked parents to report on communicative gestures, games and routine (like playing peek-a-boo), pretend play and imitation. These items were summed into a Gesture & Imitation composite variable. In addition to the CDI, questionnaires about language preferences, the child’s developmental history and family demographics were also completed by the parent.

Participants were invited to take part in an online follow-up study when the child was around 7 years. Parents provided written informed consent, and children provided verbal assent. Sessions were organised online on Teams or Zoom as per family preferences. For deaf parents, a certified Level 6 BSL user was present to facilitate communication and provide parents with access to the testing session, which took place in English between the experimenter and child. The experimenter administered assessments via the Pearson Clinical Q-Global platform. As part of a larger battery, the children were presented with the EVT-3 to assess the child’s expressive vocabulary. All bilingual children were asked to provide labels in English first and then in their other language(s) if they were bilinguals. These responses were scored by fluent users of each language and items were scored as ‘1’ if known in any language(s) and scored as ‘0’ when unknown in all languages to provide an estimate of conceptual vocabulary. When scoring in BSL, six items were flagged by fluent BSL users as not being associated with a clear BSL sign: skunk, astronaut, nostrils, duet, amphibian and chip. For these items, the BSL score was extrapolated from the mean score of the 10 closest items – five below and five higher than the excluded item. The total score was rounded to the nearest integer. The Raven’s 2 Progressive Matrices (Raven) was then administered to assess observational skills and problem solving in the non-verbal domain. Parents were invited to fill the domain-level parent/caregiver form of the Vineland Adaptive Behaviour Scales, Third Edition (‘Vineland’) to assess their child’s adaptive behaviour in the domains of communication, daily living, socialisation and motor skills.

## 3 Results

### 3.1 Group differences in early communicative skills

**Figure 1:**
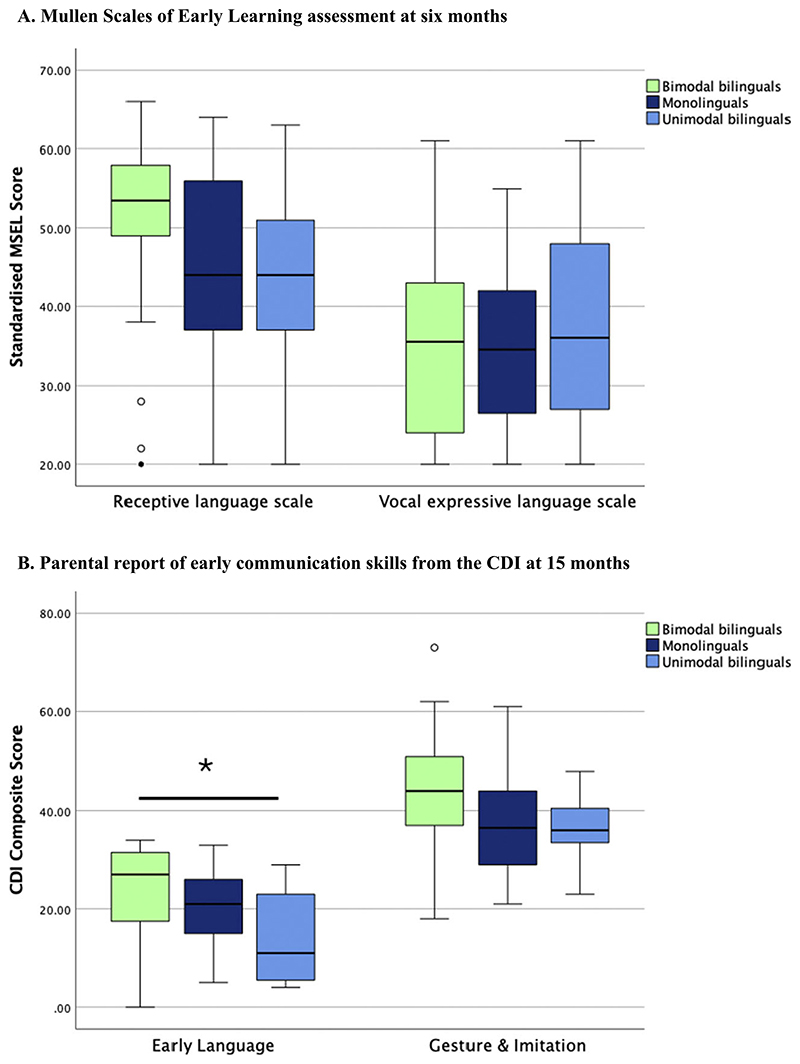
Early communicative skills measured at (A) six months via the MSEL. Standardised scores for the Receptive and Vocal expressive language scales in each group of infants with error bars displaying standard error, and (B) at 15-months via the CDI. Composite scores for items in the ‘Early words’ section (Early language) as well as in the ‘Actions & Gesture’ section (Gesture & Imitation).

At 6 months of age, early communicative skills were assessed via the receptive and the vocal expressive scales of the MSEL (see Figure 1A and details in Supplementary Material). A one-way ANOVA on the receptive language scale revealed a significant group effect [*F*(2, 91) = 3.58; *p* = .032; *η*^2^ = .08]. Bimodal bilinguals had greater receptive skills compared to unimodal bilinguals (*p* = .028), but no other group effects were observed on Bonferroni t-tests (all *p* > .30*)*. A one-way ANOVA on the vocal expressive language scale revealed no significant group effect [*F*(2, 87) = 0.44; *p* = .646; *η*^2^ = .01].

At 15 months, early communicative skills were assessed via parental report on the CDI and summarised as two composite scores: Early language and Gesture & Imitation (see Figure 1B and Supplementary Material). A one-way ANOVA with three groups was performed on Early language. Age was included as a covariate as CDI measures were not standardised for age. Gender was also added as a covariate, given a significant imbalance in gender distribution across groups at 15 months (see Table 2). A significant group effect [*F*(2, 54) = 3.26; *p* = .047; *η*^*2*^ = 0.12] and a significant effect of age [*F*(1, 54) = 5.61; *p* = .022; *η*^*2*^ = 0.10] were found. There was no significant effect of gender [*F*(1, 54) = 0.90; *p* = .348; *η*^*2*^ = 0.02]. Bimodal bilinguals significantly outperformed unimodal bilinguals on this composite measure (*p* = .008), while no other group differences were observed on Bonferroni *t*-tests (all *p* > .15). Not surprisingly, older children had better early language scores than younger children. The Gesture & Imitation composite was analysed with the same ANOVA structure. Although bimodal bilinguals numerically outperformed the other groups on the Gesture & Imitation, no significant group effects were found [*F*(2, 53) = 0.84; *p* = .440; *η*^*2*^ = 0.04]. Significant effects of age [*F*(1, 53) = 9.85; *p* .001; *η*^*2*^ = 0.23], and gender [*F*(1, 53) = 9.85; *p* = .003; *η*^*2*^ = 0.17] were observed. Gesture & Imitation scores were higher in older than younger children, and in girls than boys.

The Early Language and the Gesture & Imitation composite scores both correlated with MSEL receptive language scores at 6 months, which suggests concordance of the parental reports with behaviour observed in the laboratory setting [Early language: *r*(54) = 0.30; *p* = .026; Gesture & Imitation: *r*(53) = 0.27; *p* = .047].

### 3.2 Group differences in the development of English vocabulary

**Figure 2:**
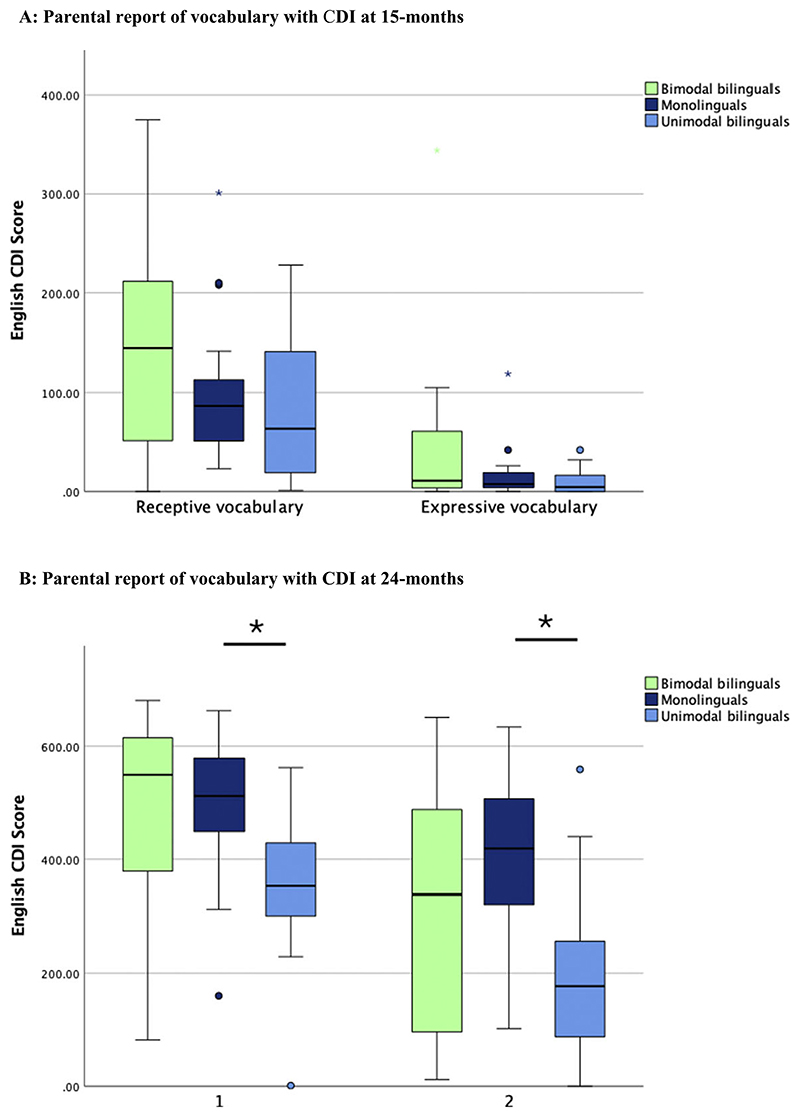
English vocabulary at (A) 15 months and (B) 24 months as assessed by parental report on the CDI vocabulary checklist.

At 15 months, the CDI vocabulary checklist was used to measure English receptive and expressive vocabulary (see Figure 2A and Supplementary Material) and analysed in a one-way ANOVA with three groups. Age was included as a covariate given that this measure was not standardised for age, and gender was also added as a covariate given a significant imbalance in gender distribution across groups at 15 months (see Table 2). No significant group difference was found for receptive [*F*(2, 54) = 1.98; *p* = .149; *η*^*2*^ = 0.075] or expressive English vocabulary [*F*(2, 54) = 2.54; *p* = .089; *η*^*2*^ = 0.09]. On both measures, a significant effect of age was found [receptive: *F*(1, 54) = 13.19; *p* = .001; *η*^*2*^ = 0.212; expressive: *F*(1, 54) = 23.16; *p* < .001; *η*^*2*^ = 0.321], while gender had no significant effect [receptive: *F*(1, 54) = 0.27; *p* = .607; *η*^*2*^ = 0.005; expressive: *F*(1, 54) = 0.36; *p* = .552; *η*^*2*^ = 0.007].

At 24 months, a one-way ANOVA with age as a covariate revealed a significant group effect for CDI-measured receptive [*F* (2, 54) = 7.44; *p* = .001; *η*^2^ = 0.23] and expressive vocabulary [*F*(2, 54) = 8.44; *p* = .001; *η*^2^ = 0.25] (see Figure 2B and Supplementary Material). In both cases, unimodal bilinguals had a smaller English vocabulary compared to monolinguals (expressive: *p* = .029; receptive: *p* = .015), while bimodal bilinguals did not differ from the other two groups (*p* > .05). On both measures, a significant effect of age was found [receptive: *F*(1, 54) = 5.07; *p* = .029; *η*^*2*^ = 0.092; expressive: *F*(1, 54) = 8.95; *p* = .004; *η*^*2*^ = 0.152], with older children having larger vocabulary scores than younger children.

**Figure 3:**
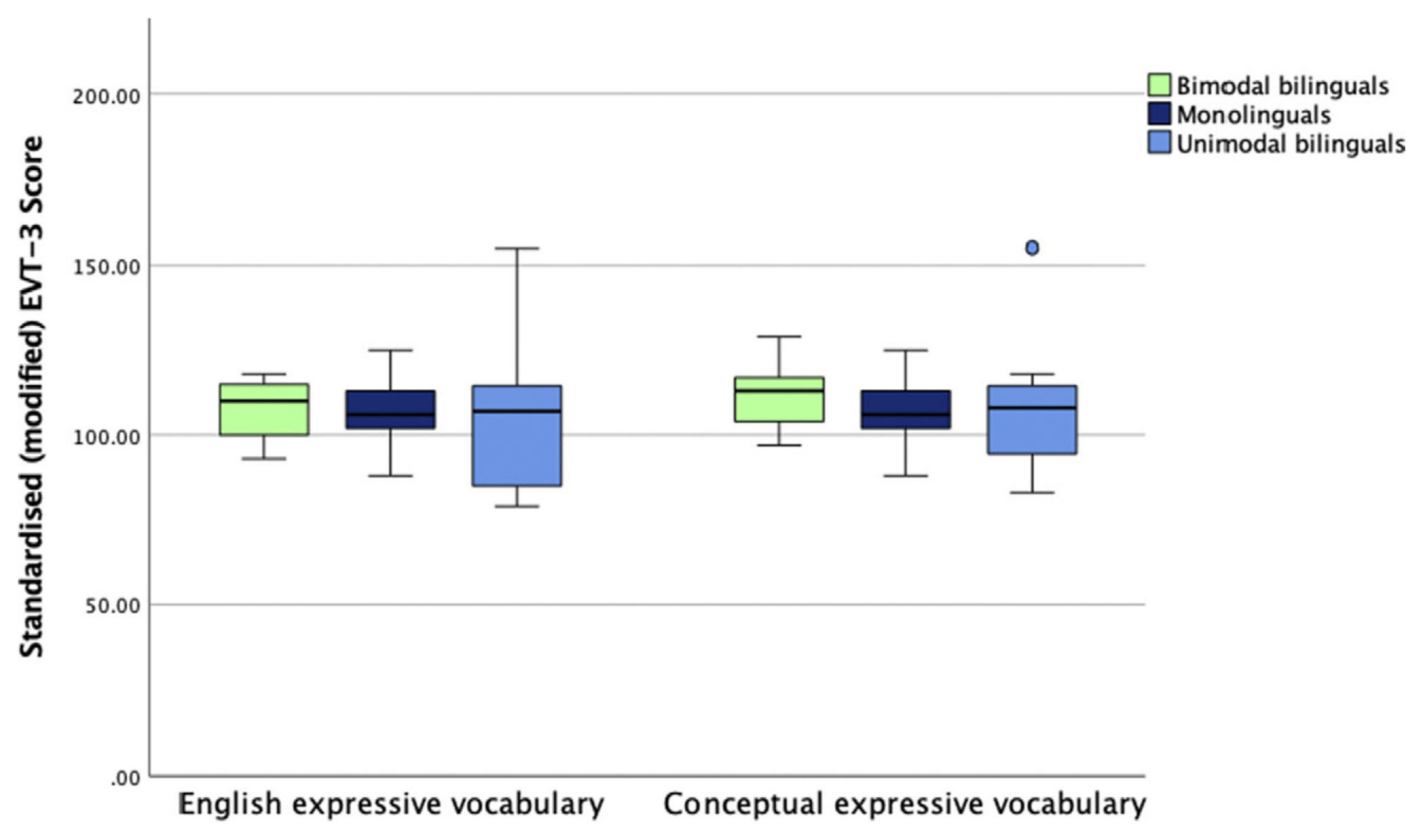
Box plot of expressive English and conceptual vocabulary measured via direct assessment with EVT-3 at 7 years.

At 7 years, the EVT-3 revealed no significant group effects on expressive English vocabulary when scored as per standard procedures (see Figure 3 and Supplementary Material) [*F*(2, 34) = 0.13; *p* = .880; *η*^2^ = 0.01]. When conceptual expressive vocabulary was scored as labels known in any language, no significant group effect emerged [*F*(2, 34) = 0.58; *p* = .567; *η*^2^ = 0.04]. There were no group differences on Vineland [*F*(2, 25) = 0.30; *p* = .745; *η*^2^ = 0.03] or Raven [*F*(2, 34) = 0.33; *p* = .725; *η*^2^ = 0.02] suggesting that the three groups of children were matched for adaptive behaviour, including communication, and problem-solving skills in the non-verbal domain. None of these analyses changed after excluding the two unimodal bilinguals receiving formal education in a non-English language. See Table 3 for a summary of group differences across time points.

### 3.3 Relationship between BSL and English vocabulary in bimodal bilinguals

To assess the relationship between BSL and English vocabulary in bimodal bilinguals, a correlation was performed between the vocabulary scores of both languages at each time point. At 15 months, a positive correlation was found between the English and BSL CDI scores for both receptive and expressive vocabulary (see Table 4). This finding suggests that bimodal bilingual toddlers rated as having a higher vocabulary in BSL also had a higher vocabulary in spoken English. At 24 months, there was a trend of a positive correlation between the BSL and English vocabulary, both receptive and expressive (see Table 4). At 7 years, the slope of the relationship between English and BSL expressive vocabulary was positive, but non-significant (see Table 4).

### 3.4 Longitudinal predictors of English vocabulary

A series of regression analyses were performed across groups to explore how concurrent and longitudinal measures of communicative development predicted English vocabulary development in a linguistically diverse group of participants. In Model 1, demographic variables that have been previously shown to reliably influence language development were entered in the model: age, gender, household income and maternal education. Model 2 tested whether a set of variables of interest had additional explanatory power. These variables were the participant’s linguistic group (monolinguals, unimodal bilinguals and bimodal bilinguals), concurrent measures of communicative development (15-months: CDI’s Early language and Gesture & Imitation composite scores; 24-months: none; 7-years: Vineland and Raven scores) and longitudinal measures of early communicative development at 6 months (receptive and expressive MSEL scores). Measures of early communicative skills at 15 months were not used as predictors of later vocabulary because of the reduced sample size due to missing data points (see detailed results in Supplementary Material).

First, a regression analysis was performed on receptive English vocabulary at 15 months. Model 1 revealed that demographic variables explained a significant proportion of variance [*R*^2^ = 0.230; *F*(4. 49) = 3.66; *p* = .011]. Age was the only significant predictor of receptive vocabulary at 15 months (*ß* = 28.71; *t* = 3.62; *p* = .001). Model 2 was a significant improvement from Model 1 [*R*^2^ = 0.746; *F*(5, 44) = 17.89; *p* < .001]. Age (*ß* = 11.74; *t* = 2.13; *p* = .039), maternal education (*ß* = 29.10; *t* = 2.45; *p* = .018) and Early language score (*ß* = 6.82; *t* = 6.99; *p* < .001) were significant predictors of receptive vocabulary at 15 months.

For expressive vocabulary at 15 months, Model 1 revealed that a significant proportion of variance was explained by demographic variables [*R*^2^ = 0.340; *F*(4,49) = 6.322; *p* < .001]. Age was the only variable that significantly predicted expressive vocabulary at 15 months (*ß* = 19.93; *t* = 4.67; *p* < .001). Model 2 was a significant improvement from Model 1 [*R*^2^ = 0.556; *F*(5,44) = 4.28; *p* = .003]. Expressive vocabulary at 15 months was significantly predicted by Age (*ß* = 11.04; *t* = 2.61; *p* = .012) and the Gesture & Imitation score (*ß* = 2.14; *t* = 3.21; *p* = .002).

At 24-months, Model 1 (age, gender, household income, maternal education) and Model 2 (demographic variables + Group, Receptive MSEL, Expressive MSEL) did not explain a significant proportion of variance in receptive English vocabulary [Model 1: *R*^2^ = 0.121; *F*(4,49) = 1.69; *p* = .169; Model 2: *R*^2^ = 0.164; *F*(3,46) = 0.79; *p* = .508] or expressive English vocabulary [Model 1: *R*^2^ = 0.115; *F*(4,49) = 1.59; *p* = .193; Model 2: *R*^2^ = 0.223; *F*(3,46) = 2.14; *p* = .108].

Finally, for expressive vocabulary in English at 7 years, a regression analysis was run with demographic variables in Model 1 (age, gender, household income, maternal education), and in Model 2, demographic variables and group, concurrent Raven and Vineland scores, receptive and expressive MSEL scores at 6 months. Model 1 and 2 did not account for a significant proportion of variance [Model 1: *R*^2^ = 0.019; *F*(4,29) = 0.14; *p* = .967; Model 2: *R*^2^ = 0.208; *F*(5,24) = 1.15; *p* = .364].

## 4 Discussion

The present study longitudinally explored the transition from early communication skills towards vocabulary development in hearing bimodal bilinguals from infancy to school-age years. Bimodal bilinguals exposed to English and BSL were compared to two different control groups – monolinguals exposed to English only and unimodal bilinguals exposed to two spoken languages. This study had four main aims: (1) to describe early communicative skills in bimodal bilinguals, (2) to assess the trajectory of vocabulary acquisition in bimodal bilinguals, (3) to test the relationship between spoken and signed language vocabulary within bimodal bilinguals and (4) to investigate how communicative skills in infancy predict vocabulary development across groups in toddlerhood and childhood.

### 4.1 Early communicative skills

Data on early communicative skills in hearing bimodal bilinguals are scarce. To clarify the earliest stages of communicative development in hearing bimodal bilinguals’ development, children were longitudinally assessed at 6 months and 15 months with commonly used measures of early development, including both direct assessment and parental report.

At 6 months, using a direct assessment, it was observed that bimodal bilinguals outperformed unimodal bilinguals in receptive communication skills. The receptive communication skills assessed by MSEL at this age are mostly social reactivity, such as engaging in a peek-a-boo game or turning around in reaction to their name. Bimodal bilinguals at 15 months were also rated by their parents as having significantly higher early language skills compared to unimodal bilinguals. The composite variable from the CDI assessed mostly the early understanding of phrases such as ‘are you hungry?’, ‘come here’ or ‘do not touch’, as well as the tendency of children to imitate expressions they had just heard or name and label objects in their environment. Parental ratings of early language skills correlated with the direct assessment of receptive language skills at 6 months as measured by the MSEL, which suggests the validity of both sets of measures.

In sum, direct assessment and parental reports jointly suggest that bimodal bilingualism may increase early communicative skills in the first and second year of life compared to unimodal bilingualism. These positive effects of bimodal bilingualism were not predicted; however, they are especially interesting given that they were observed on two different measures of early communicative skills, both designed for use with hearing children of hearing parents. It was anticipated that infants of deaf parents may be *disadvantaged* on the early communication composite measure compared to infants of hearing parents, as these measures include communicative strategies in the auditory domain that might not be used as often by deaf mothers, such as calling the infant’s name from behind their back. However, this was not the case. Having experience of interacting with both deaf and hearing communication partners may increase the infant’s awareness of communication and lead them to develop greater maturity in social communicative skills in infancy. Kanto et al. (2015) observed that hearing children with deaf parents as young as 12 months adapted their language and communication depending on the hearing status of their communication partner. They produced more signs and gestures with their deaf parent and more words with a hearing experimenter. This greater need for flexibility in communication may lead to greater maturity in communication skills measured in the receptive MSEL.

Furthermore, differences in the child’s visual attention in interactions with their mother may also contribute to the differences observed. Meadow-Orlans et al. (2004) observed that at 6 months, hearing infants with deaf mothers had more instances of looking at their mother and switched their gaze more frequently between their mother and other foci of attention than infants with hearing mothers (Meadow-Orlans et al., 2004). Given the crucial and robustly established role of visual attention in language learning (Baldwin, 2014; Tomasello, 1988), the visual demands of communicating with deaf caregivers may provide an excellent scaffold for the development of early communicative skills broadly defined.

A measure of vocal expressive language focussed on oromotor functions, vocalisations and vocal babbling was also included in the MSEL at 6 months. This scale was coded offline based on observation of child interactions. Contrary to MSEL procedures, no parental report was accepted to score any item in any of the groups. This coding procedure was adopted since many deaf parents reported not being aware of the phonological or syllabic structure of their child’s babbling. To avoid a systematic disadvantage for the bimodal bilingual group, all behaviours were scored based on vocal production displayed in a video for all infants. When scored according to this procedure, no significant differences were observed between any of the groups, which suggests that the maturity and complexity of the bimodal bilinguals’ babbling were comparable to those of monolinguals and unimodal bilinguals. This was contrary to our prediction of reduced babbling in bimodal bilingual infants, as they are likely to hear less auditory spoken language in their daily life than the other two groups of infants with hearing parents. However, reduced experience with spoken language did not result in significant differences in the maturity of babbling measured on the MSEL vocal expressive scale. Congruent with these findings, Meadow-Orlans et al. (2004) observed that maternal deafness did not negatively impact the vocal production of 6- and 9-month-old infants during a still play paradigm with their mother. Indeed, hearing infants with deaf mothers were found to produce more positive vocalisations compared to deaf infants of deaf mothers and hearing and deaf infants of hearing mothers. It is important to note that the MSEL expressive scale does not assess non-vocal forms of expressive communication used by infants, such as gesture and imitation. Given the importance of visual communication with a deaf partner, greater maturity in gesture and imitation skills can be predicted in bimodal bilinguals. These skills were assessed from parental reports in toddlerhood.

At 15 months, parents of bimodal bilinguals tended to rate their child’s use of gesture and imitation skills as numerically higher compared to the other two groups, but contrary to the prediction, this effect was not significant. It is difficult to interpret this null result, which could be attributed to a lack of power and/or to a lack of specificity of the parental reports. Indeed, Cormier et al. (1998) found that deaf infants exposed to sign language from their deaf parents tended to produce more communicative gestures than hearing infants with hearing parents, with pointing being the specific gesture that differed between groups. Sign language experience in infancy may influence the early development of some but not all gestures, and the CDI score measured here may not be specifically focussed on these. Gesture skills in bimodal bilinguals will be investigated in more detail in a separate study based on videos of parent–child interaction from the current sample.

### 4.2 Trajectory of English vocabulary acquisition in bimodal bilinguals

Vocabulary development in bimodal bilinguals has previously been described as similar to monolinguals (Bornstein et al., 1999; Brackenbury et al., 2006), better than monolinguals (Daniels, 1993), or highly variable between individuals (Hofmann & Chilla, 2015; Schiff & Ventry, 1976; Zaborniak-Sobczak, 2021). The present study measured developmental trajectories of vocabulary acquisition in bimodal bilinguals compared to monolinguals and unimodal bilinguals. Measures included both parental reports (at 15 and 24 months) and direct assessment (at 7 years). In line with effects observed in unimodal bilinguals, bimodal bilinguals were predicted to potentially have lower English receptive and expressive vocabulary compared to monolingual controls, but not compared to unimodal bilingual controls (Byers-Heinlein et al., 2024; Poulin-Dubois et al., 2013; Siow et al., 2023; Stolarova et al., 2016).

At 15 months, although bimodal bilinguals tended to have the largest receptive and expressive vocabulary sizes of all three groups of toddlers, the difference between groups was not statistically significant. At 24 months, expressive English vocabulary was significantly larger in monolinguals than in unimodal bilinguals, a finding congruent with prior literature on unimodal bilingualism (Byers-Heinlein et al., 2024; Poulin-Dubois et al., 2013; Siow et al., 2023; Stolarova et al., 2016). Bimodal bilinguals’ English vocabularies did not differ from the other two groups. While it is difficult to clearly interpret these non-significant group differences, it can be confidently stated that bimodal bilinguals were as successful at learning English vocabulary as children learning one or two spoken languages. This finding is important given that bimodal bilingualism is sometimes considered to be associated with risks in spoken language acquisition (Chen Pichler et al., 2014; St. Clair et al., 2025). The finding of equivalent English vocabulary levels between bimodal bilinguals and monolinguals is convergent with the findings of prior studies with bimodal bilinguals of various ages between 16 months and 13 years (Bornstein et al., 1999; Brackenbury et al., 2006; Daniels, 1993). It is not entirely clear why unimodal bilinguals present a difference with monolinguals in English vocabulary acquisition that is not observed in bimodal bilinguals. Bimodal bilingualism could lead to less competition between representations than unimodal bilingualism. It has previously been observed that hearing children with deaf parents have accelerated conceptual vocabulary growth in spoken English and signed ASL compared to deaf children with deaf or hearing parents (Bornstein et al., 1999). Some have interpreted this finding as an indication that access to two language modalities may be beneficial for vocabulary acquisition (Meadow-Orlans et al., 2004). Since spoken and signed languages are produced and perceived in different sensory modalities, they can be produced simultaneously using code blending (Emmorey et al., 2008). Code blending resembles co-speech gestures in its vocal-manual timing and the fact that similar meaning can be conveyed simultaneously in both communicative channels (Emmorey et al., 2008). It is possible that the production of code blending by deaf parents strengthens the learning of semantic concepts through emphasis of important words/signs in the sentence and increased attention to audiovisual productions. Further studies are required to fully test this possibility.

One potential criticism of using the CDI with multilingual children is that parents may not be fully aware of their child’s vocabulary knowledge in a language they do not frequently use with them. This applies to deaf mothers who may not have full access to the spoken language produced by their child and may interact mostly in BSL, but also to hearing bilingual parents who do not frequently use English with their child. It is difficult to fully account for this limitation while using parental report measures with bilingual children and raises the importance of confirming these findings with a direct assessment of vocabulary.

At 7 years, a direct assessment of vocabulary was completed using the EVT-3. For unimodal and bimodal bilingual children, labels were first requested in English before being requested in other languages the child was familiar with. Two vocabulary scores were computed, one standard EVT-3 English vocabulary score and one conceptual vocabulary score, which assessed whether the child knew a label for each concept in any language. There were no significant differences between groups in English and conceptual vocabulary.

In sum, bimodal bilinguals did not differ from monolingual children in their trajectory of acquisition of English vocabulary from 15 months to 7 years. Even when unimodal bilinguals were found to have smaller English vocabulary sizes compared to monolinguals, bimodal bilinguals did not differ from either group of infants with hearing parents. This suggests that bilingualism effects previously observed for unimodal bilinguals in vocabulary acquisition (Byers-Heinlein et al., 2024; Poulin-Dubois et al., 2013; Siow et al., 2023; Smithson et al., 2014; Stolarova et al., 2016; Thordardottir, 2011) may be attenuated when the two languages a child is exposed to are in different sensory modalities. Further support for this hypothesis is needed from a larger sample of unimodal and bimodal bilingual children, especially in the older time points presented here.

### 4.3 Relationship between spoken and signed language vocabulary within bimodal bilinguals

The third aim of this study was to assess the relationship between spoken and signed language vocabulary within bimodal bilinguals at 15 months, 24 months and 7 years. There was a positive slope between vocabulary in English and in BSL at each time point, which was significant at 15 months, close to significance level at 24 months, but not significant at 7 years. Although the strength of the correlation of vocabulary size in the two language modalities weakened with increasing age, these data clearly demonstrate that learning BSL does not hinder learning English. At each time point, the relationship was positive, suggesting that children who successfully learned vocabulary in BSL also tended to successfully learn vocabulary in English. The underlying source of this positive relationship is difficult to pinpoint. It could be that stronger language or general cognitive abilities positively influence vocabulary acquisition in both language modalities. Similar findings have been reported in deaf bimodal bilingual children (Pontecorvo et al., 2023). Taken together, these findings suggest that early sign language exposure does not interfere with spoken language development in hearing children of deaf parents. These data are important because many deaf mothers report facing negative attitudes and misunderstanding about the bilingual status of their child in educational and medical settings (St. Clair et al., 2025). Further data on bimodal bilingual language development could help illuminate the language profiles of those learning a signed and a spoken language, and the benefits it can offer families with deaf members.

### 4.4 Predictors of vocabulary development

The final aim of this study was to clarify the predictors of vocabulary development across the first 7 years of life in a diverse sample, including children with deaf parents. Studies with hearing children of hearing parents suggest that early communicative skills, vocal babbling and gesture are the building blocks of later language development (Donnellan et al., 2020; Goldstein et al., 2010; Matthews et al., 2018; McGillion et al., 2017; Morgan et al., 2020; Rowe & Goldin-Meadow, 2009). However, the association of these variables with later language development has not been studied in more diverse populations, including hearing children of deaf parents. To achieve this aim, regression models were run at 15 months, 24 months and 7 years with the English vocabulary size of all children as the dependent variable. First, demographic variables were entered, including the participant’s age, gender, household income and maternal education. Then a second model was run to assess whether additional variance could be explained by variables of interest. These variables included the child’s language experience group, as well as concurrent and longitudinal measures of communication skills at 6 months.

At 15 months, receptive vocabulary was significantly predicted by the participant’s age and maternal education. Concurrent parental rating of early communication score explained additional variance in receptive vocabulary at 15 months. Expressive vocabulary was predicted by the infant’s age as well as by concurrent parental rating of gesture and imitation. No significant predictor was associated with vocabulary size at 24 months and at 7 years. The relatively small sample size at these time points could have contributed to these non-significant findings.

Age was a significant predictor of vocabulary size at 15 months (receptive and expressive). In both cases, older children had larger vocabulary sizes than younger children. This is not surprising given that these measures were not standardised for age, and that the age range of the sample was quite wide. On the other hand, EVT-3 was standardised for age and the regression analysis did not reveal a significant effect of age on this measure of vocabulary at 7 years. Linguistic group, gender and socio-economic variables were not significant predictors of vocabulary at any time point, except for a significant impact of maternal education on receptive vocabulary at 15 months.

Unlike in previous literature with children of hearing adults (Morgan et al., 2020), receptive communication skills did not appear to contribute longitudinally to vocabulary acquisition in a diverse group of infants, including unimodal and bimodal bilinguals. Indeed, receptive MSEL score at 6 months was not a significant predictor of vocabulary at any of the later time points collected. It remains possible that these skills could contribute to other aspects of language and communication, such as communication skills and pragmatics. Based on prior literature with children of hearing adults, vocal babbling, gesture and imitation skills were expected to be significant predictors of longitudinal vocabulary development (Donnellan et al., 2020; Goldstein et al., 2010; Matthews et al., 2018; McGillion et al., 2017; Rowe & Goldin-Meadow, 2009). While vocal babbling maturity at 6 months did not have any predictive value for later developing vocabulary, parental ratings of gesture and imitation maturity were a significant predictor of concurrent expressive vocabulary at 15 months. These findings suggest that the transition towards the first words may be more heavily anchored in communicative skills such as gesture and imitation than on vocal babbling maturity in a sample including children exposed to sign language.

#### 4.4.1 Limitations and considerations for future studies

There are several limitations to the present study, which need to be kept in mind when interpreting the data and planning future studies. The first limitation is the sample size. While the starting sample in infancy represents one of the largest published on this population, significant attrition occurred in longitudinal time points. The non-significant results in the later time points, especially at 7 years, where the sample was smallest, could result from a lack of power. These analyses call for a replication with a larger sample.

A second potential limitation of the present study comes from the way bilingualism was defined. This study considered children to be bilingual if they had ‘frequent and regular exposure to more than one language’. There is currently no consensus in the literature about the definition of bilingualism (Byers-Heinlein, 2015), but many studies consider a child to be bilingual if they have at least 20% exposure to their non-dominant language (e.g., DeAnda et al., 2016). The bilinguals in the present study sometimes had more imbalanced exposure, including, for example, a child exposed to 98% BSL and 2% English. In the sample of the current study, 5 out of 31 bimodal bilinguals and 5 out of 27 unimodal bilinguals would not have been considered bilingual under a ‘20% exposure criterion’. This broad definition of bilingualism was adopted in the present study to reflect the realities of hearing children of deaf parents who vary greatly in their exposure to each of their signed and spoken languages. Since this population is very rare, setting strict exposure criteria would have prevented the recruitment of a large sample. For consistency, the same definition of bilingualism was also used to recruit unimodal bilinguals. This broad definition could have contributed to the large interindividual variability observed in the bilingual groups, especially on English-specific measures, such as vocabulary. Moreover, the unimodal bilinguals in the current sample include children exposed to various languages in addition to English. It has been clearly demonstrated that unimodal bilingual acquisition is influenced by the linguistic distance between their languages (Blom et al., 2020; Floccia et al., 2018; Havy et al., 2016; Sundara & Scutellaro, 2011). Since it is impossible to match bimodal bilinguals and unimodal bilinguals in linguistic distance, the unimodal bilingual group included children exposed to various languages. This approach also has the advantage of limiting the effects of cultural and socio-economic disparities between the monolingual and a specific bilingual group by adding more variability in the bilingual group. However, prior research suggests that vocabulary acquisition could differ in groups of bilinguals uniformly exposed to closer versus further languages (Blom et al., 2020; Floccia et al., 2018). This is important context for interpreting group differences involving the unimodal bilingual group in the present study. Finally, at the 7-year time point, the unimodal bilingual sample included two children receiving formal education in their non-English language, but the result of analyses remain unchanged after excluding these children. All findings in this study would benefit from a replication with a sample of bilinguals with more homogeneous language profiles.

A third limitation of the present study is the wide within-group age range and imperfect matching of the groups in terms of demographic and socio-economic characteristics. Indeed, age ranges were wide at each time point, but there were no significant differences across groups. While the MSEL, EVT-3, Raven and Vineland scores take age into account in their standardised scores, the raw CDI scores do not. For this reason, age was entered as a covariate in analyses of group differences on CDI scores. Not surprisingly, age was a significant covariate, with larger scores observed in older children. However, age did not change the significance of the group effects observed. Moreover, the unimodal bilingual group included more boys than other groups, which led to a significant imbalance in gender distribution across groups at 15 months, but not at other time points. However, gender was not a significant covariate of vocabulary or early communicative skills at this age, so we can be confident that the results in this age group would have been similar if groups were matched for gender. Socio-economic factors are another important point to keep in mind in studies of bilingualism (Byers-Heinlein, 2015; Golinkoff et al., 2019). In the present study, despite a similar level of education, deaf mothers were less represented than hearing mothers in high-income categories. Unfortunately, this sample reflects the reality of the United Kingdom, where deaf adults are likely to be overrepresented in lower-status, lower-paid posts compared to hearing adults (Shield, 2019). Despite this mismatch in socio-economic status across groups, and the robustly demonstrated impact of such differences on early language development (Golinkoff et al., 2019), the only group differences found in the present study between bimodal bilinguals and other groups were positive in direction. This suggests that even larger advantages could be predicted in groups perfectly matched for socio-economic status.

A final potential limitation of the present study comes from the unavailability of standardised tools to flexibly assess bilinguals, and especially bimodal bilinguals. For this reason, standard procedures were adapted to assess the sample of children included in this study. These adaptations, described in detail in the methods section, included (1) ruling out parental report from the MSEL expressive language scale, (2) using the English and BSL CDI to assess vocabulary and (3) creating a non-standardised conceptual vocabulary measure using EVT-3. The conceptual vocabulary score presented here was based on an adaptation of a test designed for application in English and was not normed in each language. The BSL version of the task may have been mismatched with the other spoken language tasks. It could be that the specific BSL signs assessed in EVT-3 had a different age of acquisition, frequency or a phonology that made them easier/harder to produce than the equivalent words in some of the other languages. It is also possible that the coding of responses had a different leniency in BSL than in spoken languages. These measures are imperfect and stress the importance of continuing to develop language assessment tools in multiple languages, including signed languages.

## 5 Conclusions

Taken together, the results of the present study suggest a healthy picture of vocabulary development in bimodal bilinguals from 6 months to 7 years. There was no evidence of sign language exposure harming or interfering with spoken language development. Instead, bimodal bilingualism may lead to strengths in early communicative skills. In recent years, the increasing amount of data on unimodal bilingualism has allowed a shift in perspective. Learning two spoken languages is now better understood as a language learning trajectory that does not represent two monolingual speakers in one body, a trajectory that is not categorically better or harder than monolingualism, and one that differs greatly between bilingual individuals (Genesee, 2016). A similar change in perspective is now required in the field of bimodal bilingualism. The data presented here suggest that bimodal bilingualism should not be viewed as a question of speech versus sign, with one language modality taking away from the other, but as an adaptive language learning profile. Not only is it possible for bimodal bilinguals to acquire vocabulary at a similar rate to monolingual English speakers, but the knowledge of sign language is also likely to be vital to establish an optimal parent–child relationship and access to the Deaf culture.

## Figures and Tables

**Table 1:** Summary of the literature assessing vocabulary development in hearing children with deaf parents. Studies are organised per age according to the youngest child in the sample. Blank cells indicate unassessed skills. Merged cells for spoken and signed vocabulary suggest that studies allowed children to answer in any modality without differentiating them in analyses

Study reference	Sample size	Age range	Measure	Spoken vocabulary finding	Signed vocabulary finding
Schiff and Ventry (1976)	*n* = 52	6 mo to 12 years	Informal interaction with examiner + formal assessments including Peabody Picture Vocabulary Test (PPVT)	*n* = 10 of *N* = 52 have vocabulary or other language deficit unexplained bydeafnessor other factors	
Meadow-Orlans et al. (2004)	*n* = 15	12 months	Free play observation	10 of 15 have at least one recognisable sign/word – similar to children of hearing adults	
Brackenbury et al. (2006)	*n*=1	16 mo, 20 mo	Communication Development Inventory (CDI) in English and ASL	Comparable to monolinguals	Comparable to deaf native signers
Bornstein et al. (1999)	*n* = 21	17–30 mo	Early Language Inventory; Reynell Expressive and Comprehension Scales	Comparable to hearing children of hearing adults	
Daniels (1993)	*n* = 14	2-to–13 years	PPVT	Significantly higher than monolinguals	
Hofmann and Chilla (2015)	*n*=6	3-to–6-years	Standardised assessments, including PDSS to assess vocabulary + unstandardised assessment of spoken German for multilingual children (HAVAS 5)	6 of 6 below monolinguals on vocabulary for verbs 5 of 6 within average range on a tool designed for bilinguals	
Zaborniak-Sobczak (2021)	*n*=6	4-to–8 years	Language Development Test	Receptive: 2 of 6 below monolingual norms Expressive: 4 of 6 below monolingual norms	

**Table 2:** Sample size and descriptive statistics of infants’ age and gender at each time point. [Maximum, minimum]; mths = months; yrs = years; M = mean; Std Dev = Standard deviation

	Bimodal bilinguals	Monolinguals	Unimodal bilinguals	Demographic differences across groups
6-months	*n* = 31 (18 girls)	*n* = 33 (16 girls)	*n* = 27 (8 girls)	Gender: *x*^2^(2) = 4.81; *p* = .090
	[4.3–8.7 mths]	[4.3–7.9 mths]	[3.9–7.7 mths]	Age: *F*(2, 91) = 0.36, *p* = .698
	*M* = 6.4 mths	*M* = 6.2 mths	*M* = 6.2 mths	
	*Std Dev* = 1.2	*Std Dev* = 0.9	*Std Dev* = 1.0	
15-months	*n* = 20 (12 girls)	*n* = 22 (8 girls)	*n* = 12 (1 girl)	Gender: *x*^2^(2) = 9.52; *p* = .014
	[13.0–19.4 mths]	[13.4–20.0 mths]	[13.8–16.3 mths]	Age: F(2, 54) = 0.07; *p* = .930
	*M* = 15.2 mths	*M* = 15.0 mths	*M* = 15.1 mths	
	*Std Dev* = 1.6	*Std Dev* = 1.6	*Std Dev* = 0.9	
24-months	*n* = 18 (10 girls)	*n* = 21 (10 girls)	*n* = 15 (5 girls)	Gender: *x*^2^(2) = 1.65; *p* = .438
	[24.0–33.4 mths]	[23.3–34.6 mths]	[23.4–34.7 mths]	Age: F(2, 54) = 0.63; *p* = .538
	*M* = 26.4 mths	*M* = 26.3 mths	*M* = 27.4 mths	
	*Std Dev* = 3.2	*Std Dev* = 3.2	*Std Dev* = 3.2	
7 years	*n* = 10 (5 girls)	*n* = 13 (6 girls)	*n* = 11 (5 girls)	Gender: *x*^2^(2) = 0.05; *p* = .975
	[6.7–8.9 yrs]	[6.5–8.1 yrs]	[6.7–8.1 yrs]	Age: F (2, 34) = 0.99; *p* = .382
	*M* = 7.8 yrs	*M* = 7.5 yrs	*M* = 7.4 yrs	
	*Std Dev* = 0.7	*Std Dev* = 0.5	*Std Dev* = 0.6	

**Table 3:** Summary of group differences across measures and time points. M = Monolingual; UB = Unimodal bilingual; BB = Bimodal bilingual. Effects presented describe significant Bonferroni corrected post hoc tests following a significant Group effect in the main ANOVA analysis. NULL indicates no statistically significant Group effects. Shaded cells indicate measures not taken at that time point

	Timepoints			
	6 months	15 months	24 months	7 years
Early communication				
Receptive language (MSEL)	BB > UB			
Vocal expressive language (MSEL)	NULL			
Early language (parent-report CDI)		BB > UB		
Gesture & Imitation (parent-report CDI)		NULL		
Vocabulary				
English receptive vocabulary (parent-report CDI)		NULL	M > UB	
English expressive vocabulary (parent-report CDI)		NULL	M > UB	
English expressive vocabulary (EVT–3)				NULL
Conceptual expressive vocabulary (Modified EVT–3)				NULL

**Table 4:** Summary of within-group correlations between English and BSL vocabulary in bimodal bilingual children

	English receptive	English expressive
BSL receptive	15mo: *r*(17) = 0.82; *p* < .001	–
	24mo: *r*(14) = 0.49; *p* = .054	
BSL expressive	–	15mo: *r*(17) = 0.54; *p* = .018
		24mo: *r*(14) = 0.46; *p* = .074
		7 yrs: *r*(9) = 0.33; *p* = .384

## Data Availability

Data are available with the permission of research participants by contacting Evelyne Mercure (https://www.gold.ac.uk/psychology/staff/mercure-evelyne/).

## Highlights

- This paper studied hearing children with deaf parents exposed to BSL and English.
- These children are experiencing bimodal bilingualism.
- Bimodal bilinguals show a similar trajectory of vocabulary acquisition to monolinguals.
- Bimodal bilingual infants have better communicative skills than unimodal bilinguals.
- Bimodal bilinguals show a positive correlation between BSL and English vocabulary.

## Footnotes

**Supplementary material.** The supplementary material for this article can be found at http://doi.org/10.1017/S1366728925100308.
